# Negotiating the postvention situation: A grounded theory of NHS staff experiences when supporting their coworkers following a colleague’s suicide

**DOI:** 10.1080/07481187.2023.2297056

**Published:** 2024-01-10

**Authors:** Johanna Spiers, Hilary Causer, Nikos Efstathiou, Carolyn A. Chew-Graham, Anya Gopfert, Kathryn Grayling, Jill Maben, Maria van Hove, Ruth Riley

**Affiliations:** a School of Health Sciences, University of Surrey, Guildford, UK; b Institute of Applied Health Research, University of Birmingham, Birmingham, UK; c School of Medicine, Keele University, Keele, UK; d Department of Health Life Sciences, University of Exeter, Exeter, UK; e NHS Employers, Leeds, UK; f Department of Health Life Sciences, The University of Exeter- Saint Lukes Campus, Exeter, UK

## Abstract

Suicide is a leading cause of death. NHS workers, especially female nurses, have heightened vulnerability. Being impacted by a colleague’s suicide can lead to increased suicidality. Postvention refers to support following a suicide. We investigated current, available postvention for NHS workers following a colleague’s suicide and the experiences of staff who deliver it (“supporters”). Twenty-two supporters were interviewed, and data were analyzed using classic grounded theory. The theory of negotiating postvention situations was developed. Supporters must negotiate enabling and disabling elements that form a “postvention situation” and impact behaviors and postvention efficacy. Postvention delivery is emotionally burdensome. Supporters need support, which they do not always receive. Postvention can lead to learning, which can better inform future postvention. The extent to which NHS workers can effectively support colleagues will depend on their postvention situation. As such, work must be done to enable supporters to offer effective postvention in the future.

Suicide; postvention; healthcare workers; grounded theory

Suicide is a leading cause of death, globally, with more than 700,000 people dying by suicide every year (World Health Organisation, 2021). In the UK, NHS doctors, female nurses, and male paramedics are more likely to die by suicide than their counterparts in the general population (NHS Employers, 2023). Effective postvention can help improve outcomes for those who have lost someone to suicide (Hill, 2021; Lestienne et al., 2021). However, while scholars have developed various postvention models for clinicians who have lost a patient or client to suicide (Becker et al., 2017; Castro et al., 2021; Gutin, 2019; Leaune et al., 2020; Quinett, 2009), until recently (Kinman & Torry, 2021; Samaritans & NHS Confederation, 2023), there has been little postvention guidance for NHS colleague suicide available. Additionally, what did exist was often not evidence-based (Causer et al., 2022). NHS staff are an at-risk population, vulnerable to chronic stress, anxiety, depression, vicarious trauma, and burnout (Health Education England, 2019). This means they may not be best placed to manage the impact of colleague suicide nor offer additional caring responsibilities. Few researchers have explored the impact of delivering postvention; however, authors of a recent review demonstrated complex pressure on managers who were tasked with postvention delivery following a colleague suicide (“supporters”; Causer et al., 2022). Thus, we set out to investigate the mechanisms and impact of delivering postvention to NHS workers following a colleague’s suicide.

## Method

### Design and ethics

To our knowledge, there is currently no theory related to postvention delivery within healthcare settings. We used grounded theory, which creates substantive theories that are grounded in data (Birks et al., 2019) and so can inform the design of interventions for practitioners and researchers (Starks & Trinidad, 2007). This paper is part of a larger study on the impact on, and support needs of, NHS staff following a colleague’s suicide. Our sister paper (in preparation) explores the impact of colleague suicide on staff.

We suggest that much existing suicide research pathologizes suicide as an individualized mental health problem (Causer et al., 2022). We adopted a critical suicidology lens to consider the complex cultures and contexts within which supporters operate and suicides happen (White, 2017).

Ethical approval for this study was given the University of Birmingham (ERN_20-1566) and HRA (IRAS 291050).

### Participants

Twenty-two supporters participated. These participants were NHS workers who self-identified as having supported their colleagues following the suicide of a coworker, either because this was part of their role (if they were chaplains or wellbeing leads) or because they were managers to whom the work fell. Participants were not postvention specialists. Demographic information is in Table 1.

**Table 1.** Participant demographic information.

Participant Characteristics		
Gender	Male	4
	Female	18
Ethnic identity	White British	21
	British African	1
Age range		25–65 years
Job role	Nurse	2
	Nurse manager	9
	Non-clinical manager	1
	Doctor	2
	Wellbeing lead	3
	Chaplain	5
Time since suicide occurred		6 months–10 years
Numbers of suicides experienced	One	14
	Two	7
	Three or more	1

We recruited participants via Participant Identification Centers (PIC) at several NHS Trusts in England. We also utilized social media and snowball sampling. Once theoretical sampling began, we directly contacted several NHS chaplains.

### Procedure and analysis

Once potential participants had consented and completed a demographic questionnaire, we set a date for a telephone or online video interview. Interviews were undertaken by JS (n = 15), HC (n = 6), and RR (n = 1). The topic guide included questions on the relationship with the person who died by suicide, their reaction to the death, and the experience of supporting others and being supported (or not). All interviews were digitally recorded, transcribed, and anonymized by professional transcribers.

Data collection and analysis took place simultaneously. Nine interviews were analyzed by JS, using classic grounded theory (Glaser & Strauss, 2017). Transcripts were coded for behavioral incidents which illustrated conceptual categories. Constant comparison was used to revise codes so that they best represented participants’ core concerns. Memos were used to record analytic thinking about concerns and behaviors. Codes and memos were used to develop the theory (Glaser & Strauss, 1967).

Early analysis informed later interviews (Glaser & Strauss, 2017). For example, in response to early codes and memos, JS asked subsequent participants about the capacity their team had to handle their workload at the time of the suicide. After analyzing 14 interviews, theoretical sampling was used to recruit more participants (Birks et al., 2019). Specifically, we sought supporters who did not know the person who died by suicide, as we were interested in exploring whether this influenced their ability to deliver postvention. Additionally, existing participants tended to report delivering good support, so we sought supporters who had experienced challenges in delivering postvention. However, we were unable to recruit participants to meet this criterion.

**Figure 1.**
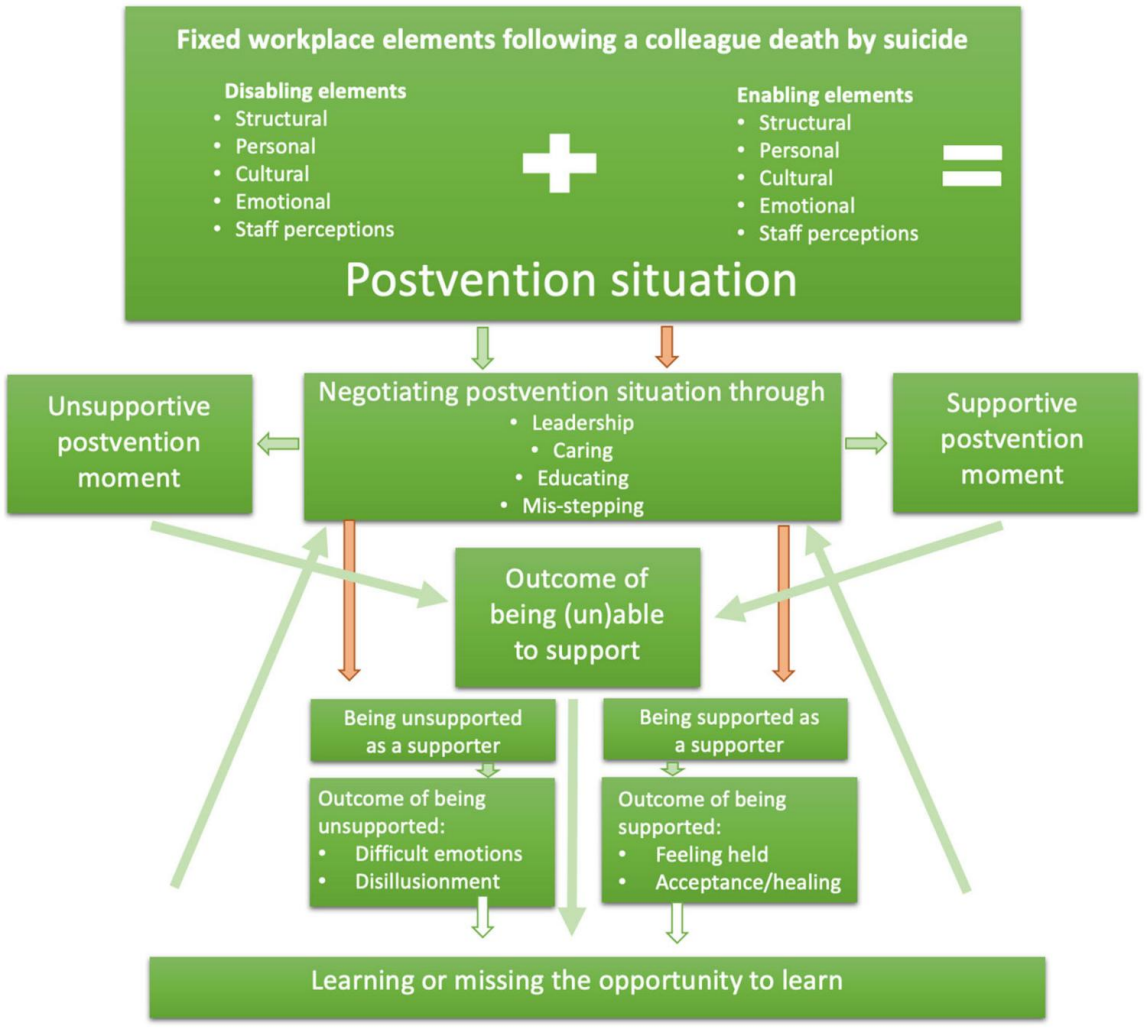
Negotiating postvention situations: a grounded theory.

Analysis continued while the final seven supporter participants were recruited. Once all analysis was completed, and theoretical saturation was achieved (Low, 2019), the theory of “Negotiating postvention situations” was developed (Figure 1).

## Results

Figure 1 illustrates the theory of “Negotiating postvention situations”, demonstrating how the elements which comprise a supporter’s postvention situation inform the behaviors they can choose to engage in, which lead to the delivery of supportive or unsupportive postvention. Delivering postvention has an emotional outcome for supporters. All of this may lead to learning which can improve future postvention, meaning that the negotiation of the postvention situation is a cyclical process. Additionally, supporters themselves need support that may or may not be forthcoming. Being supported, or not, will have an emotional outcome. The green arrows indicate the situation/behaviors/outcomes of the supporters, whereas the orange arrows refer to the situation and behaviors of those supporting the supporters.

### Fixed workplace elements create supporters’ postvention situations

NHS workers who support their colleagues following a coworker’s suicide must do so from a contextual situation comprised of enabling and disabling elements. These elements, which supporters cannot control, create a unique “postvention situation” which defines and/or restricts their ability to deliver support, be supported, and learn.

#### Disabling elements

Disabling elements make it harder for a supporter to deliver postvention. These might be structural; cultural; personal (such as a supporter’s level of experience); emotional (how the suicide has emotionally impacted the supporter); or staff perceptions (beliefs held by staff that make delivering postvention harder).

While structural and cultural factors as well as staff perceptions are clearly fixed aspects of the postvention situation, personal and emotional elements might be argued to be within a supporter’s control. Individuals are, of course, responsible for their job roles and the years of experience they have acquired. However, when it is discovered that a colleague has died by suicide, supporters cannot decide to have more experience, nor can they switch role. Similarly, supporters may feel affected by the suicide. Thus, all elements of the postvention situation are usually out of a supporter’s control.

Structural disabling elements reported by participants include having no capacity. This could be physical (no space to sit and grieve); staff-related; financial; or connected to the structure of the service – for example, those working in 9–5, Mon–Fri wellbeing teams cannot support staff out-of-hours: “everybody can see us and they can check in. But they [busy team] are isolated behind their doors, so there is no good time to capture them” (N27).

Many participants reported that their entire trust had a lack of capacity: “You need to be able to let people have that breathing space. […] But nobody’s got it because we’re on this treadmill of the contract and the KPIs [key performance indicators]” (SS04). Additionally, teams of workers constantly shift with the NHS, meaning it can be hard for supporters to reach all colleagues who may have been impacted by a suicide.

Most participants reported a lack of training or postvention guidance. Further, issues of confidentiality restricted supporters. For example, if a family did not consent to share that the death was a suicide, this could hinder postvention.

Supporters may also work in blaming, unrelenting, hostile, or static cultures. Stigma about suicide specifically and mental health in general were often reported. Again, all these elements hinder effective postvention.

Personal disabling elements included a lack of experience in individuals: “we don’t have the expertise in place, we can’t rely on one clinical psychologist who may or may not be available” (N27).

Disabling emotional elements included supporters’ reactions following a colleague suicide. Shock, sadness, anger, and regret were reported. Supporters may also have to juggle their own grief with postvention: “I didn’t wanna go off sick […] I wanted to be there with the team in it but I just also I was just sitting at my desk staring at a screen crying” (N24).

If supporters have experienced the death, suicide, or suicide attempt of several colleagues, the compound effect may make postvention harder: “even though my rational mind was saying you’re not personally responsible, […] I really felt the weight of that. […] And it knocked my confidence” (SS12).

Additionally, staff’s perceptions of supporters could act as a disabling element. For example, a chaplain reported that NHS workers “won’t even look at me” (SS09) due to (incorrect) preconceptions that chaplains only deliver religious care. Additionally, one wellbeing lead (SS12) felt that coming to see her might be perceived as stigmatizing.

#### Enabling elements

Enabling elements make it easier for a supporter to deliver effective postvention. These can also be structural, cultural, personal, or emotional factors or staff perceptions.

Enabling structural elements included having enough capacity within a team, helped by adequate staffing. Teams that include chaplains, psychologists, wellbeing leads, and managers have a better capacity for dealing with postvention.

Participants shared that useful guidance or training enables postvention. While specific postvention training was only mentioned by one, some had access to trauma, wellbeing, or psychological training/guidance which could be adapted for postvention.

A caring culture also enabled postvention efforts. Care manifested through relationships, communications, meetings, or actions. Additionally, an enabling culture can adapt, for example, by shifting from management leadership to compassionate leadership.

Not just putting the corporate political spin out there […] But actually, backing it up and being prepared to have the tough conversations […] the organization that I work for […] has come on leaps and bounds, and I think if this [suicide] happened now, it would be a completely different story. (N08)

Personal enabling elements relate to supporters’ experience and role. Role seniority, expertise in mental health and suicide, or experiential/professional learning derived from having lived through the suicides of other staff or family members were helpful. Various job roles were more suited to delivering postvention. For example, wellbeing leads and chaplains perceived themselves to be well-positioned for postvention: “we [chaplains] can do the listening, the supporting, […] the creating a memorial service […] and then […] if we do all that and somebody is still struggling, then we signpost them” (SS08).

Several supporters who did not know the person who died felt they personally did not need support. This also may be an enabling element as it might facilitate some distance.

Staff can also have enabling perceptions of supporters. For example, staff may be more likely to turn to supporters they consider friends, those who make conversations about mental health more acceptable, or those who are familiar: “If you keep showing your face, and people start going oh you know they’re alright, […] even if you’re associated with bringing biscuits” (SS10).

One wellbeing lead reported that staff were more likely to come back for further support after an initial individual session, suggesting that building trust and psychological safety were key: “we know once people have used this [wellbeing] service, they’re much more likely to come back and use it again” (SS12).

### Negotiating the postvention situation

Supporters negotiate their unique postvention situation by engaging (or not) in various behaviors. These are leading, caring, educating, and mis-stepping.

#### Leading

Supporters engaged in a variety of leadership behaviors to deliver postvention. Successful communication about suicide required leadership. The need to deliver the news quickly and in an emotionally congruent fashion was reported:

I said ‘you know what I’m like, usually I’m all about happy and everything but right now there’s no way I can dress this up. What I’m gonna tell you is shit. […] You’re gonna be sad, I’m feeling really sad, but I’ve got to tell you now’. (N02)

Supporters need to acknowledge the death rather than remain silent about it. Additionally, they sometimes needed to control the news by ensuring it was rolled out in the best way possible, such as by highlighting their strong social media policy.

The ability to take charge and act immediately was beneficial. Supporters with seniority or mental health expertise felt able to take charge, switch into ‘professional mode’ and thus offer psychological safety to colleagues in the uncertain aftermath of suicide. However, this might make supporters more vulnerable to harm.

I had to do all that sort of manager mode if you know what I mean. […] Had to do the process and then think of the staff. […] at that time […] you can’t always think of your own feelings, that comes later […] You have to keep face and do. (N11)

Some supporters engaged in ‘carrying on’; that is, helping the team or themselves to get back to ‘business as usual’; a perceived necessity in high-demand, patient-facing work.

#### Caring

Supporters who engaged in caring behavior employed emotional labor, offering empathic and compassionate responses. Examples included taking affected colleagues out for lunch, making efforts to contact all affected colleagues (including those who had moved on to a new team), and legitimizing colleagues’ feelings. Supporters who are aware of their staff’s individual needs (an enabling element) could tailor postvention:

…we did go and [break the news to] another colleague just before that meeting. And this is a lady whose [family member] very tragically took her life […] she said ‘I won’t come into that meeting now thank you for telling me individually’. (SS04)

Several supporters reported on activities that contained colleagues’ difficult emotions by offering psychological safety or enabled emotional authenticity. Activities included offering reflective spaces for chatting “organically and naturally” (SS06) about feelings. Offering availability by being vocal about the support on offer was also beneficial.

Some supporters engaged in self-care. Reported examples included self-reflection, exercise, asking for support, and establishing boundaries.

#### Educating

Several supporters used psychoeducation to teach staff about suicide or signs of mental ill health. Some fought stigma by talking about suicide or acknowledging the death without judgment. Others pushed for changes to hospital policies and procedures: “I went to the director of people within the NHS […] And then I went to [chief nursing officer] […] And I said we need to do something about this” (N25).

#### Mis-stepping

The disabling elements that supporters negotiate in their postvention situations create barriers to providing good enough postvention. For instance, silence around a suicide (perhaps underpinned by stigma) resulted in an absence of support:

…so it was never sort of mentioned to myself as wellbeing lead, that […] [name] had ended his life through suicide. […] and so no formal processes were put in place. And so we didn’t go out and make sure that we were visible. (N27)

Participants reported support plans that had to be abandoned due to red tape or a lack of capacity. Other missteps might be linked to not knowing what to do: “it was just left there and no one really knew how to deal with it. So you know, cause no one had an idea it wasn’t dealt with” (N19).

In contrast, doing too much could also be a misstep: “Often there’s a bit of a race to go out and firefighting, do some early support. And the worst thing people - is to have loads people going in” (N01).

The helpful and unhelpful behaviors that are engaged in as a response to the postvention situation create a range of “postvention moments”. These are individual instances of the postvention that are delivered to, and experienced by, affected staff.

### Postvention moments

Support must be sustained to be effective. It must be on offer promptly, and for an extended period, so affected colleagues can access it when they need it. However, postvention is made up of individual moments that staff might interpret as either supportive or unsupportive. We theorize that a supporter might deliver moments that are experienced as supportive by some staff and unsupportive by others.

#### Supportive postvention moments

Whilst postvention support must be adaptive to respond to complex needs and contexts following a suicide, postvention tools can be simple. Talking and listening were frequently cited. Affected colleagues need to be able to gather in a group so that they can talk and share stories about the person who died by suicide; supporters need to listen, either to groups of staff or in one-to-one sessions.

One chaplain described how a memorial activity enabled colleagues to talk about the person who died by suicide: “That seemed to be quite cathartic. Everyone then talked about what have you done [to memorialize the person who died by suicide]? Oh I remember him doing that. What have you done? Oh yeah, I’ve done that” (SS09).

The power of talking was demonstrated by the many participants who commented during or after the interview that it was good to talk about the person who had died: “it was nice talking about [name] actually, I can picture her again” (SS03).

While impacted colleagues need to come together to talk, supporters perceived their job was to listen: to be able to sit, non-judgmentally, with another’s grief, whether in group or individual settings. For example: “Int: What was it that you were providing during that time? SS01: A listening ear more than anything. A reflective, responsive, supportive presence I think I would say” (SS01).

Togetherness was a key aspect of postvention. Supporters who had the resources provided time for colleagues to be together in a group safe space. Supportive postvention also included practicalities such as time off work and shifts being covered.

Effective postvention moments also had an emotional element. Supporters emphasized the importance of congruency; being present with the difficult emotions suicide creates. Additionally, supportive postvention might trigger a release of emotion. Affected colleagues may need to be able to cry or express frustration, anger, or shock: “when we hear shocking news, it’s almost like we go “Oh!” and hold our breath. So, for me, the debrief is just about starting to breathe out” (SS06).

Supporters also facilitated the emotional process of making sense of the death by sharing professional knowledge about suicide.

Remembrance is another key aspect of supportive postvention. This involves recalling the person who died by suicide through stories or memorializing them through (for example) gardens or donations. Participants gave examples of supporting colleagues to attend funerals or facilitating memorial services.

One participant shared how supportive postvention also operated as prevention by empowering staff members to voice suicidal thoughts and access support: “a staff member had killed themselves and then others […] were suicidal, so I then contacted their line manager to say ‘you have a team that is really struggling’ and then we got psychological services and wellbeing involved” (SS10).

Suggestions for this preventative element of postvention included asking staff if they were having suicidal thoughts; finding out new recruits’ mental health needs; and access to more resources. This final suggestion demonstrates that having more enabling elements (e.g., greater resources) in a postvention situation may lead to better postvention, thus saving lives.

#### Unsupportive postvention moments

If supporters are unavailable, postvention will be ineffective. Supporters might be unable to help due to (e.g.,) a lack of capacity. Unsupportive postvention moments might also include a lack of emotional congruency, silence, or uncertainty about the suicide: “the implication is that he died by suicide but no one […] has ever confirmed or denied that and no one’s been allowed to talk about that” (D11).

Remembrance which colleagues do not feel sufficiently represents the person who died or their feelings about the death could also be seen to be damaging. One chaplain said:

People need to feel safe; they need to know that somebody’s in charge […] And you can feel the difference, if you get somebody leading a service who can’t do that, you will feel the anxiety in the congregation. (SS08)

Support must be sustained and available over a short, medium, and longer timeframe. Delays are not supportive. While some participants were unable to support staff immediately after the suicide for structural reasons (e.g., the suicide happening at a weekend), one was unable to access talking therapy for herself for six months: “I got referred to [company] through work but they said they can’t assist with grief for six months, post […] death” (N24). By the time she could access this support, she no longer felt she needed it.

### Outcomes of being (un)able to offer support

Delivering postvention had an emotional outcome for supporters. Whilst some outcomes only appear to come from delivering supportive postvention and others from unsupportive postvention, there was also some crossover.

Delivering supportive postvention led to satisfaction for some supporters and appreciative feedback from staff. For example, when one participant ran a postvention session for their colleagues, they said staff “grabbed it with both hands” (N08). However, the emotional costs of offering postvention were more challenging. These included emotional exhaustion, anxiety, sadness, and isolation: “Everyone thinks that I’m very kind of calm and get on with things. But they don’t really know about the stuff inside” (SS09).

Delivering supportive postvention can also lead to trauma and burnout, or growth and learning: “if anything it’s give me a kind of strength […] there’s kind of a spiritual component to my response to it” (N01).

Supporters are operating from a fixed postvention situation which, at times, means they cannot provide the postvention or culture they would like. Yet they carry the frustration, anger, professional doubt, and moral injury that arises from being unable to deliver supportive postvention or make a long-term difference following the death. Here are two examples: “my own sense of, you know, am I actually up to the job (scoffs). You know, am I the right person to be doing this” (SS12) and “we’d sort of (pause) promised ourselves and promised [the deceased’s] family we would make a difference. And (pause) and it didn’t feel like we really had much to show for it” (D02).

These outcomes demonstrate the need for supporters themselves to be supported. Delivering supportive postvention is complex and challenging; it is not its own reward.

### Being (un)supported as a supporter

Supporters shared that, like staff, they needed support when a coworker died by suicide. For a more in-depth look at the impact of suicide and the support needs of impacted colleagues, read our sister paper (in preparation). However, these NHS workers also need support in their supportive roles.

Support for supporters might come from managers and supervisors. Importantly, those who are being asked to offer compassion and empathy to the supporters should be a good fit for the role. Having supportive and reliable teammates is also beneficial: “there was me and the senior nurse, I think and we did it [offering support] together” (N26).

Supported supporters felt held and accepted, expressed admiration for their colleagues, and moved toward healing.

Provision of support for supporters can be undermined by the narrative that ‘carers don’t need care’ within organizational cultures (Conolly et al., 2022). Also, supporters can only be supported if there is capacity. One explained that they were often the only chaplain on shift, so if something difficult happened, they were unsupported.

Reported features of poor postvention for supporters include blame and an absence of support: “I reached out to everybody and offered it to them but nobody actually offered it to me” (M01).

Some supporters cited difficulty in accepting support, which might come from the internalization of the ‘carers don’t need care’ narrative or perceptions that others weren’t interested in helping. A lack of support for supporters led to upset, anger, a sense of abandonment, and burnout.

I think that’s part of the reason I ended up burning out. Because a lot of it I just thought I’ll just say I’m fine cause that’s easier than the reality because I didn’t feel they were interested anyway. (N19)

Some disillusioned supporters felt empowered to speak truth to power, informing managers that the provision of support was inadequate and needed improvement.

### Learning

All postvention experiences could lead to learning, helping supporters to provide better postvention, in the present and future. Learning can take several forms. Supporters in this study reported more postvention and professional skills as well as increased awareness of mental health issues and their own boundaries and needs.

Teams or trusts might also learn from postvention. For example, gaps in service might be spotted. One supporter found that their team became more open to talking about mental health.

…it also probably made our […] hospital executive team sign up to the idea that wellbeing is important. […] It is really difficult to make it properly meaningful in a massive organization. But there definitely was a willingness rather than it not being important and I think [the deceased] contributed to that. (D02)

Any formal learning or evaluation activities must be carried out with care, involving those who were close to the situation. One participant related that a factually inaccurate report about a suicide in which they had been a supporter damaged their mental health.

…this report had been written without any consultation with myself or anybody […] who knew [the person who died by suicide] […] I felt it was worded in such a way it felt like it was trying to apportion some blame? […] which really is very disturbing and making [me] very very unhappy. (M01)

When done well, learning can change policy. Examples from participants included a better approach to complaints about healthcare professionals, time off for funerals, and more wellbeing resources in general. Several participants who had delivered postvention without guidance had contributed to writing their own: “[my colleague] and I developed the post-incident psychological support service, which is for […] all staff following any sort of traumatic incident, but generally that’s suicide, death of a colleague” (N01).

Some participants also reported that opportunities to learn had been missed, perhaps due to stigma or a lack of capacity, demonstrating how disabling elements of a postvention situation can impact postvention delivery all the way to the final stage of learning.

When I asked for a suicide plan to be put onto the agenda, the response was ‘we’ll talk about it but I don’t want the words suicide plan put on the agenda. We’ll call it any other business’. And I found that bizarre, because why are we not talking about this? (N27)

## Discussion

NHS workers who deliver postvention following a colleague suicide are operating within a combination of enabling and disabling elements (their postvention situation), which makes their ability to deliver effective postvention, via various behaviors, easier or harder. Whether postvention is supportive or not, its delivery has an emotional impact on supporters which is often damaging yet can also result in satisfaction or growth. All postvention can lead to learning for individuals and employers.

Our theory of negotiating postvention situations demonstrates that, like suicide (Marzetti et al., 2022), postvention does not happen in a vacuum. While each suicide will depend on individual factors impacting the person who dies by suicide, structural contributors must be included when considering prevention (Marzetti et al., 2022). We argue that the same is true for postvention. Since effective postvention can act as prevention (Andriessen, 2009), all potentially relevant elements must be accounted for when delivering postvention support. Critical suicidologist White (2017) has argued that, for example, rather than suicidal queer people having to re-assess heterosexist violence, violence itself should be addressed. Similarly, we suggest that rather than those who struggle to offer supportive postvention in a disabling situation having to re-assess their own worth or abilities, the systems themselves need to change. This systemic approach to postvention is novel since critical suicidologists have, thus far, only critiqued the pathologization and individualization of suicide itself rather than postvention (Marzetti et al., 2022; White, 2017).

Given the above, a key question is how the elements of a postvention situation can be facilitated to ensure they are enabling. Supporters must have the capacity to offer postvention and be supported themselves. This is a challenge in the NHS, where many professionals, including GPs (Spiers et al., 2017), junior doctors (Riley et al., 2021), and nurses (Palmer, & Rolewicz, 2022) are leaving the profession due to poor working conditions. We found that supporters who worked in an “enabling role” (i.e., wellbeing leads and chaplains) tended to have more resources for postvention than managers. Therefore, we recommend utilizing workers in these roles when delivering postvention. Further, a lack of training or guidance inhibits the delivery of postvention. Some guidance in this area now exists (Kinman & Torry, 2021; Samaritans & NHS Confederation, 2023) and evidence suggests that training is beneficial (Clements et al., 2022). Hence, we propose that evidence-based training and guidelines, including information on how to lead, care and educate when supporting affected colleagues, be offered to NHS workers who may be expected to deliver postvention.

Our findings demonstrate the influence that personal elements such as experience and expertise can have on postvention. Enabling personal elements might include being in a wellbeing/chaplaincy role; seniority/years of job experience; mental health expertise; and professional or personal experience of suicide. Given the variation in this list, postvention should ideally be delivered by a trained team of NHS workers, rather than solitary professionals (Business in the Community Public Health England, & Samaritans et al., 2017; Causer et al., 2022; Samaritans, 2021). The utilization of such a team may also enable trust in those staff being supported following a suicide and mean that staff with different support needs can approach different people for support.

Stigma leads to inadequate postvention (Causer et al., 2022). It is harder for supporters to deliver postvention if they feel unable to discuss suicide at work. Many authors reporting on colleague suicide have stated that the death was not properly acknowledged (Berkowitz et al., 2011; Carr, 2011; Deheegher, 2008; Lynn, 2008), including in healthcare settings (Kleespies et al., 2011; Samaritans, 2021). Given that talking about what happened and being together to share grief and memories appear to lead to effective postvention, we encourage a culture in which mental health and suicide can be discussed, as this helps to challenge stigma (Clements et al., 2022; Rivart et al., 2021; Samaritans & NHS Confederation, 2023) and provide better support.

Additionally, we found that their emotional response to the suicide may impact NHS workers’ ability to deliver postvention. Quantitative researchers may wish to explore any links between the level of grief a supporter is feeling and their ability to support others.

Remembrance is another key aspect of postvention. Various authors suggested caution here, with some advising against the erection of permanent memorials to those who have died by suicide (Business in the Community, Public Health England, & Samaritans, 2017; Samaritans, 2021; Samaritans & NHS Confederation, 2023). However, we have found no evidence to underpin this advice. Given the positive feedback that many of the supporters in this study had from conducting memorializing activities – including creating memorial items – we suggest that this area needs further investigation.

Supporters also talked about the need to “carry on” their team’s patient-facing work despite bereavement. This narrative is potentially harmful (Causer et al., 2022), yet manager supporters are often in an impossible situation where patient care delivery must continue despite grief. Further research is needed to address this thorny issue.

Several supporter participants either reported that they could not access talking therapy right away or felt concerned that support which was offered too soon would not be effective. While we searched the literature for confirmation that talking therapies following bereavement should not be offered too quickly, we couldn’t find any evidence to support this idea, although it may have grown out of the suggestion that one-off debrief sessions following trauma have the potential for long-term negative outcomes (Rose et al., 2003). We suggest that effective postvention – which differs from bereavement counseling – needs to be delivered in a timely, sustained manner, so that as many sessions of support are available for those who need it, when they need it.

Given that many NHS workers will be delivering support from a disabling postvention situation, is it unsurprising that many will mis-step, offering inadequate or non-existent support. Our sister paper on the experiences of NHS staff who have lost a colleague to suicide illustrates this (in preparation). Given that NHS supporters are working from a postvention situation that is largely out of their control, we propose that better resourcing and education are more useful responses to a lack of support than blame.

Delivering postvention in a work setting, including the NHS, has an emotional impact (Causer et al., 2022). Therefore, guidance and training need to include information about how to care for the carers, as well as impacted staff.

Finally, given that all postvention may lead to learning, we propose it should always be evaluated and results used to improve future support (Gulliver et al., 2016), something which currently does not happen often (Causer et al., 2022).

### Strengths and limitations

This is the first study to investigate the experiences of, and impact on, NHS workers who offer postvention to colleagues. It also presents the first theorization of enabling and disabling elements and contexts in the delivery of postvention support. Given that NHS workers are vulnerable to suicide (NHS Employers, 2023), we suggest that this is important work and that our novel use of a critical suicidology lens (Marzetti et al., 2022; White, 2017) is necessary since supporters are operating from a postvention situation they cannot control.

Notwithstanding these strengths, this study has limitations. Firstly, despite our efforts to connect with NHS workers from ethnically diverse populations, our sample was almost exclusively white British. Given that Black and minority ethnic people comprise almost a quarter of that workforce (NHS England, 2023), this means our findings are unlikely to represent the experiences of all staff members. We suggest that future researchers engage with community gatekeepers to mitigate similar problems (Renert et al., 2013). Secondly, most participants in this study were delivering supportive postvention. Although we proactively sought participants who were delivering less optimal support, we could not recruit these people. Thus, the paper reflects the views and experiences of those providing supportive postvention and may give the impression that NHS workers are more fully supported following the suicide of a colleague than in reality. We direct readers to our sister paper (in preparation), in which many colleagues impacted by suicide describe the impact of a considerable lack of support. However, we believe that the views and experiences of the participants in this study can provide informative learning opportunities for trusts who wish to deliver effective postvention.
